# MOVIN PAST OBSTACLES: BUILDING A CULTURE OF MOBILITY

**DOI:** 10.1093/geroni/igad104.0193

**Published:** 2023-12-21

**Authors:** Sarah Glowinski, Mary Hook, Barbara King

**Affiliations:** Advocate Health, Milwaukee, Wisconsin, United States; Advocate Health, Milwaukee, Wisconsin, United States; University of Wisconsin-Madison, Madison, Wisconsin, United States

## Abstract

Older adults are at risk for losing their ability to ambulate independently during hospitalization. The “MOVIN” (Mobilizing Older adult patients Via a systems-based Intervention) Study is a cluster-randomized control trial aimed to implement a nursing-led mobility model and monitor the change in nurse behaviors and unit culture before, during and after the 14-week intervention period. The model includes psychomotor training, mobility resources (equipment and ambulation aide), communication tools, ambulation pathways, and support for developing a “culture of mobility” led by nurses. It was launched on two inpatient nursing units at large urban Magnet quaternary medical center in Wisconsin in July 2021 (Unit 1) and March 2022 (Unit 2). Electronic reports based on documentation were generated to monitor patient ambulation and distances for feedback and celebration. The researchers and unit teams met often to implement and ensure intervention integrity despite increasing care demands and staffing shortages. Unit leaders navigated pandemic-induced obstacles including turnover, shortages, and high patient volumes by refining staff training, streamlining communication, promoting in-room walking, and finding creative ways to celebrate and build a culture within the COVID-19 restrictions. Unit 1 ambulated 75% of patients (20% increase) with distances averaging 47 miles/month (540% increase). Unit 2, with less ambulatory patients, ambulated 73% of patients (18% increase) with distances averaging 23.4 miles/month (680% increase). MOVIN is a promising system-based intervention that supports culture change and promotes nurse-led patient ambulation. Units were able to increase and sustain the quantity and quality of patient walking despite the obstacles imposed by the pandemic.

